# Evaluation of the Antiviral Activity of Sephin1 Treatment and Its Consequences on eIF2α Phosphorylation in Response to Viral Infections

**DOI:** 10.3389/fimmu.2019.00134

**Published:** 2019-02-12

**Authors:** Maxime Fusade-Boyer, Gabriel Dupré, Pierre Bessière, Samira Khiar, Charlotte Quentin-Froignant, Cécile Beck, Sylvie Lecollinet, Marie-Anne Rameix-Welti, Jean-François Eléouët, Frédéric Tangy, Barbora Lajoie, Stéphane Bertagnoli, Pierre-Olivier Vidalain, Franck Gallardo, Romain Volmer

**Affiliations:** ^1^Université de Toulouse, ENVT, INRA, UMR 1225, Toulouse, France; ^2^Viral Genomics and Vaccination Unit, CNRS UMR-3569, Institut Pasteur, Paris, France; ^3^NeoVirTech SAS, Institute for Advanced Life Science Technology, Toulouse, France; ^4^UMR 1161 Virology, INRA, ANSES, Ecole Nationale Vétérinaire d'Alfort, ANSES Animal Health Laboratory, EURL for Equine Diseases, Maisons-Alfort, France; ^5^UMR INSERM U1173 2I, UFR des Sciences de la Santé Simone Veil—UVSQ, Montigny-le-Bretonneux, France; ^6^AP-HP, Laboratoire de Microbiologie, Hôpital Ambroise Paré, Boulogne-Billancourt, France; ^7^Unité de Virologie et Immunologie Moléculaires (UR892), INRA, Université Paris-Saclay, Jouy-en-Josas, France; ^8^Laboratoire de Génie Chimique CNRS, INPT, UPS Université de Toulouse III, Faculté des Sciences Pharmaceutiques, Toulouse, France; ^9^Laboratoire de Chimie et Biochimie Pharmacologiques et Toxicologiques, Equipe Chimie & Biologie, Modélisation et Immunologie pour la Thérapie, CNRS UMR 8601, Université Paris Descartes, Paris, France

**Keywords:** PKR, GADD34, PPP1R15A, virus, antiviral, eIF2α, host, broad-spectrum

## Abstract

The guanabenz derivative Sephin1 has recently been proposed to increase the levels of translation initiation factor 2 (eIF2α) phosphorylation by inhibiting dephosphorylation by the protein phosphatase 1—GADD34 (PPP1R15A) complex. As phosphorylation of eIF2α by protein kinase R (PKR) is a prominent cellular antiviral pathway, we evaluated the consequences of Sephin1 treatment on virus replication. Our results provide evidence that Sephin1 downregulates replication of human respiratory syncytial virus, measles virus, human adenovirus 5 virus, human enterovirus D68, human cytomegalovirus, and rabbit myxoma virus. However, Sephin1 proved to be inactive against influenza virus, as well as against Japanese encephalitis virus. Sephin1 increased the levels of phosphorylated eIF2α in cells exposed to a PKR agonist. By contrast, in virus-infected cells, the levels of phosphorylated eIF2α did not always correlate with the inhibition of virus replication by Sephin1. This work identifies Sephin1 as an antiviral molecule in cell culture against RNA, as well as DNA viruses belonging to phylogenetically distant families.

## Introduction

Most clinically available antiviral drugs act by directly targeting viral components to inhibit a critical step in the viral life cycle, such as entry, replication, or viral egress (1). These molecules have several advantages, as they can be very potent inhibitors and should have minor side effects because they are, in theory, virus specific. However, viruses evolve constantly and the selective pressure of the treatment can give rise to mutants that are resistant to these drugs. This is illustrated for example by the emergence of influenza virus strains resistant to viral neuraminidase inhibitors (2).

By contrast, antiviral molecules targeting host functions that are necessary for the virus life cycle are less likely to lead to the emergence of resistant viral mutants (3). Moreover, broad-spectrum antiviral molecules can be developed if the targeted host cell function regulates the replication of a wide range of viruses. Numerous host factors have been identified as required for viral replication through whole-genome genetic screens, providing impetus to develop antiviral molecules targeting these host factors (4). The numerous pathways experimentally identified as potential targets for antiviral therapy include viral entry or egress, viral assembly, viral protein synthesis or maturation, and the immune response against viruses (3, 5, 6). Currently, approved drugs targeting the host include the widely used type I interferons, which boost the antiviral innate immune response, ribavirin, which modulates the pool of intracellular nucleosides and is reported to modulate the innate immune response, and finally maraviroc, inhibiting human immunodeficiency virus entry by targeting C-C chemokine receptor type 5 (CCR5) (1). In an effort to limit toxicity, it is necessary to target a host cell function that is not crucial to the cell physiology and/or that is more specific to infected cells.

The phosphorylation of serine 51 of the α subunit of eukaryotic translation initiation factor 2 (eIF2α) inhibits initiation of protein translation in response to various cellular stresses (7). Four protein kinases have been shown to specifically phosphorylate eIF2α. The protein kinase RNA-like endoplasmic reticulum kinase (PERK) phosphorylates eIF2α in response to endoplasmic reticulum stress, due to the accumulation of unfolded proteins in the endoplasmic reticulum or to perturbations of the endoplasmic reticulum membrane lipid composition (8, 9). The haem-regulated inhibitor kinase (HRI) phosphorylates eIF2α in response to iron deficiency and has been demonstrated to regulate the differentiation of red blood cells (10). The general control non-derepressible-2 (GCN2) phosphorylates eIF2α in response to amino-acid deficiency (11). Finally, the interferon-induced double-stranded RNA-activated protein kinase (PKR) phosphorylates eIF2α in response to the accumulation of viral RNA harboring a double-stranded or other nucleic acids secondary structures produced during viral replication (12). Increased eIF2α phosphorylation attenuates translation of most mRNAs and is a physiological response to adapt to the various cellular stresses described above. Activation of PKR is for example an antiviral response aiming at reducing the translation of viral proteins in infected cells. The importance of PKR in antiviral defense is illustrated by the broad-array of viral countermeasures selected during evolution to inhibit PKR activation or eIF2α phosphorylation (12). It should however be noted that increased eIF2α phosphorylation seems to benefit to some viruses, including viruses belonging the *Togaviridae* family (13), *Reoviridae* family (14), and hepatitis C virus (15), most likely because translation of their mRNAs relies on secondary structures from which initiation can proceed even in the presence of high levels of eIF2α phosphorylation (12). As a consequence, developing means to increase eIF2α phosphorylation could be an antiviral intervention only for viruses whose mRNA translation is inhibited by increased eIF2α phosphorylation.

Dephosphorylation of eIF2α allows the cell to resume initiation of protein translation and is achieved by a binary complex between the catalytic phosphatase subunit PP1 and a regulatory subunit composed of either GADD34 (or PPP1R15A) (16) or CReP (or PPP1R15B) (17). The regulatory subunits GADD34 and CReP target the phosphatase PP1 specifically to the phosphorylated eIF2α substrate. CReP is constitutively expressed. By contrast, GADD34 expression is induced by eIF2α phosphorylation and therefore should be specifically expressed in stressed cells. GADD34 thus provides a negative feedback on eIF2α phosphorylation (8).

The guanabenz derivative Sephin1 was shown to increase eIF2α phosphorylation in cells stimulated with drugs causing PERK activation via the accumulation of unfolded proteins in the endoplasmic reticulum lumen (18). Sephin1 was described as a specific inhibitor of GADD34, although the identity of its target is currently subject of debate [see section Discussion and (19–21)]. We reasoned that inhibition of GADD34 could have antiviral effects by potentiating eIF2α phosphorylation in infected cells. Moreover, given that GADD34 is induced in cells with increased eIF2α phosphorylation, a GADD34 inhibitor should specifically act in stressed cells, such as infected cells, thus enhancing drug selectivity.

In the current work, we provide evidence that Sephin1 exhibited antiviral effects against specific viruses belonging to various viral families. In addition, Sephin1 increased eIF2α phosphorylation in response to activators of PKR, suggesting that Sephin1 may act by increasing eIF2α phosphorylation in virus-infected cells.

## Materials and Methods

### Reagents and Cellular Treatments

Cells were treated for 16 h with 2.5 μg/ml tunicamycin (Sigma, USA) or with 1 μg/ml of intracellularly delivered Poly(I:C) (HMW)/LyoVec (Invivogen, France). Sephin1 was purchased from Tocris (United-Kingdom) or synthesized according to the protocol described in Das et al. (18). Purity was verified by nuclear magnetic resonance. Sodium arsenite (Sigma, USA) was added to cells in culture at a final concentration of 500 μM for 1 h before lysis. Cells were treated for 24 h with 1,000 U/ml of bacterially produced recombinant human interferon α A (PBL assay science, USA).

### Cells and Viruses

Human HEK293, HEK293T, human ARPE-19, and rabbit RK13 cells were grown at 37°C in DMEM containing glutamate supplemented with 10% FBS, 1x penicillin-streptomycin. Human HEp-2 cells were grown at 37°C in MEM containing glutamate supplemented with 10% FBS, 1x Penicillin-Streptomycin. Wild-type mouse embryonic fibroblasts (MEF WT) and MEF in which the endogenous eIF2α gene has been genetically replaced by a nonphosphorylable (S51A) allele (MEF S51A) have been described previously and were kindly provided by David Ron, University of Cambridge, United Kingdom (22, 23). Human respiratory syncytial virus (hRSV), derived from the strain Long, genetically modified to express firefly luciferase or the fluorescent protein mCherry were previously described and used to infect HEp-2 cells (24). Enterovirus D68, kindly provided by Caroline Tapparel, Université de Genève, Switzerland (25), was used to infect human RD cells cultured at 33°C, as previously described (26). Human adenovirus serotype 5 (hAdV), belonging to serotype 5, genetically modified to express the bacterial partitioning system-based AnchOR3 was used to infect human HEK cells, as recently described (27). Measles virus strain Schwartz genetically modified to express the firefly luciferase (28) was used to infect human HEKT cells, as previously described (29). Myxoma virus strain T1 was used to infect RK13 cells as previously described (30). Human cytomegalovirus (hCMV) derived from the TB40/E strain and genetically modified to express the bacterial partitioning system-based AnchOR3 was used to infect human ARPE-19 cells, as recently described (31). The AnchOR3 system is distributed by NeoVirTech SAS, France and is available upon request. Influenza A/Puerto Rico/8/1934 (H1N1) and A/turkey/Italy/977/1999(H7N1) were used to infect A549 or MDCK cells, as previously described (32, 33). Japanese encephalitis virus genotype 3 strain Nakayama (34) was used to infect HEK293T cells. Briefly, HEK293T cells were infected with JEV at a MOI of 0.01 for 48 h and JEV RNAs in cell supernatants were quantified by real-time RT-PCR as described in Yang et al. (35). Theiler's murine encephalomyelitis virus (TMEV) genetically modified to express a mutant L protein and the fluorescent protein Cherry (36) was used to infect MEF WT and MEF S51A. Cellular viability was measured with Vita-Blue Cell Viability Reagent (Biomake) according to the manufacturer's protocol. This assay is based on a fluorescent dehydrogenase enzymes substrate, which correlates with cellular metabolic activity.

### Western-Blot Analyses

Cells were lysed as previously described (37) and used for western-blot analyses. Phosphorylated eIF2α was detected with a polyclonal rabbit antibody (ab32157, Abcam, United-Kingdom) or (44-728G, ThermoFischer Scientific, USA). Total eIF2α was detected with a polyclonal rabbit antibody (Proteintech, USA).

### Quantification of Virus Replication

Myxoma virus titers were determined by standard plaque assay on RK13 cells, as described in Camus-Bouclainville et al. (30). Enterovirus D68 and influenza virus titers were determined by the tissue culture infectious dose 50 (TCID_50_) method, as described in Soubies et al. (38). We measured replication of luciferase expressing virus 24 h post-infection by lysing cells and measuring light emission on a Clariostar (BMG Labtech) plate reader using the Luciferase assay System kit (Promega) according to the manufacturer's instructions. We measured replication of TMEV expressing the fluorescent protein Cherry by measuring fluorescence using a Clariostar (BMG Labtech) plate reader. hRSV expressing Cherry was detected in paraformaldehyde fixed HEp-2 cells by immunofluorescence and imaged using a confocal microscope. Replication of hAdV and hCMV expressing AnchOR3 protein was quantified by measuring GFP foci using automated microscopy, as described in Komatsu et al. (27) and Mariamé et al. (31).

### Rabbit Infections and Treatments

Rabbit infections and treatments were described in a protocol approved by the Ethical committee Science et Santé Animale (SSA 115) and the French Ministry of Research (protocol reference number 2015112009419390). Rabbits were infected by injection of 50 plaque-forming units of myxoma virus wild-type strain in the dermis of the right ear. The myxoma virus wild-type strain LH 3082 used for the *in vivo* infection was isolated in 2008 from the eyelid of a rabbit found dead in a farm in the South West of France. Sephin1 was solubilized in DMSO at a concentration of 1 mg/ml and further diluted in pineapple juice to administer either at 5 mg/kg (first experience), or 100 mg/kg (second experiment) by a single daily oral administration. Control animals received equivalent volumes of DMSO in pineapple juice. Animals were monitored daily for clinical signs and conjonctival swabs were performed at the indicated days post-infection to monitor for virus replication as recommended (39).

## Results

### Consequences of Sephin1 Treatment on eIF2α Phosphorylation in Cells Stimulated With Known Stimulators of eIF2α Kinases

**Figure 1:**
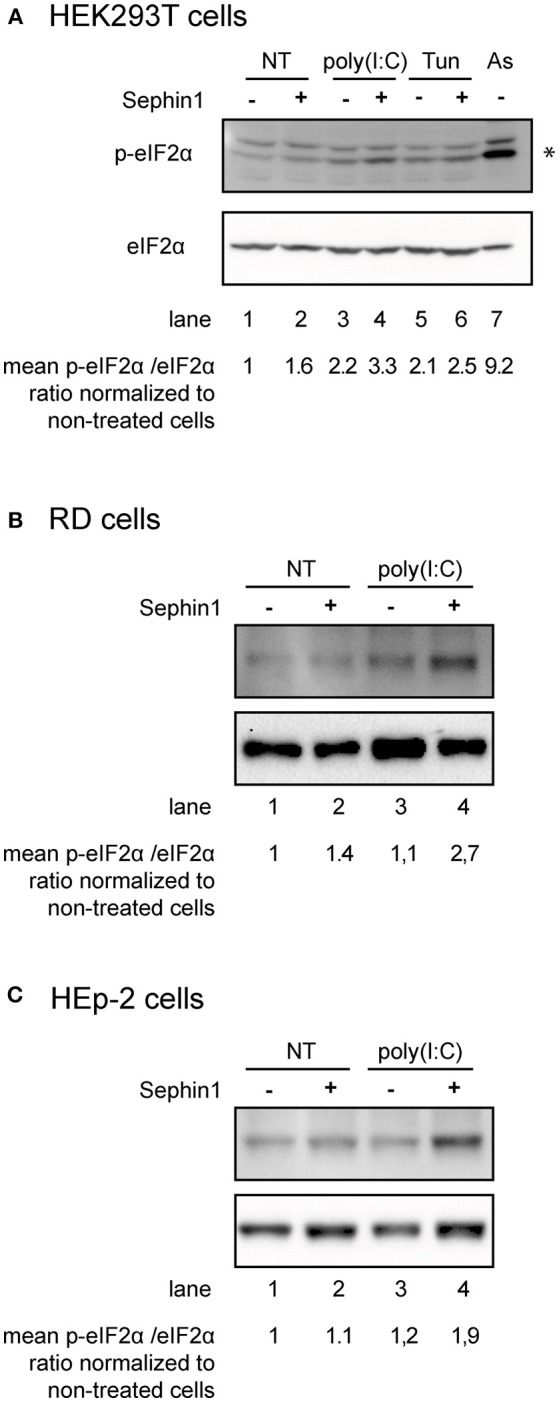
Consequences of Sephin1 treatment on eIF2α phosphorylation. **(A)** HEK293T cells were either left untreated (NT), treated for 16 h with intracellularly delivered poly(I:C), which stimulates PKR or with tunicamycin (Tun), which stimulates PERK, in the presence or absence of 50 μM Sephin1. The asterisk indicates the position of phosphorylated eIF2α, as revealed in cells stimulated with sodium arsenite (As), a potent inducer of eIF2α phosphorylation. **(B)** RD cells and **(C)** HEp-2 cells were either left untreated (NT) or treated for 16 h with intracellularly delivered poly(I:C), in the presence or absence of 50 μM Sephin1. The mean fold increase of the phosphorylated eIF2α phosphorylation/total eIF2α ratio normalized to non-treated cells calculated from three independent experiments is shown below the photographs.

To determine the levels of eIF2α phosphorylation, we performed western-blot analysis using antibodies against phosphorylated eIF2α and against total eIF2α. In order to verify the specificity of these antibodies, we treated cells with sodium arsenite, a well-known potent inducer of eIF2α phosphorylation that mainly activates HRI (40). High levels of phosphorylated eIF2α were detected in sodium arsenite treated cells (Figure 1A, lanes 7), demonstrating the specificity of these antibodies and the position of the band corresponding to phosphorylated eIF2α, indicated with an asterisk. To evaluate the consequences of Sephin1 treatment on eIF2α phosphorylation, we exposed HEKT cells to the glycosylation inhibitor, tunicamycin, a known inducer of ER stress causing the accumulation of unfolded proteins in the ER. The accumulation of unfolded proteins in the ER leads to the activation of PERK, which phosphorylates eIF2α. As expected, eIF2α phosphorylation was increased in cells treated with tunicamycin (Figure 1A, lanes 1 vs. 5). Co-treatment with Sephin1 increased tunicamycin-induced eIF2α phosphorylation (Figure 1A, lanes 5 vs. 6), in agreement with previously published results (18). We next evaluated if Sephin1 could also potentiate eIF2α phosphorylation in the context of viral infections by stimulating cells with intracellularly delivered Poly(I:C), a synthetic RNA mimicking viral RNA and known to stimulate PKR (41). Poly(I:C) induced eIF2α phosphorylation (Figure 1A, lanes 1 vs. 3), which was further increased in cells treated simultaneously with Sephin1 (Figure 1A, lanes 3 vs. 4). Upon interferon α-pretreatment, which is known to upregulate PKR expression, we observed increased eIF2α phosphorylation in Poly(I:C)-treated HEK293T cells (Supplementary Figure 1, lanes 3 vs. 7). Simultaneous treatment with Sephin1 further increased eIF2α phosphorylation in Poly(I:C)-treated cells (Supplementary Figure 1, lanes 7 vs. 8). Sephin1 treatment also increased eIF2α phosphorylation in RD cells (Figure 1B) and in HEp-2 cells (Figure 1C) treated overnight with intracellularly delivered Poly(I:C). Altogether, these results suggest that Sephin1 could boost PKR-mediated eIF2α phosphorylation, possibly by inhibiting GADD34-mediated dephosphorylation of eIF2α.

### Evaluation of the Antiviral Properties of Sephin1 Against RNA Viruses

**Figure 2:**
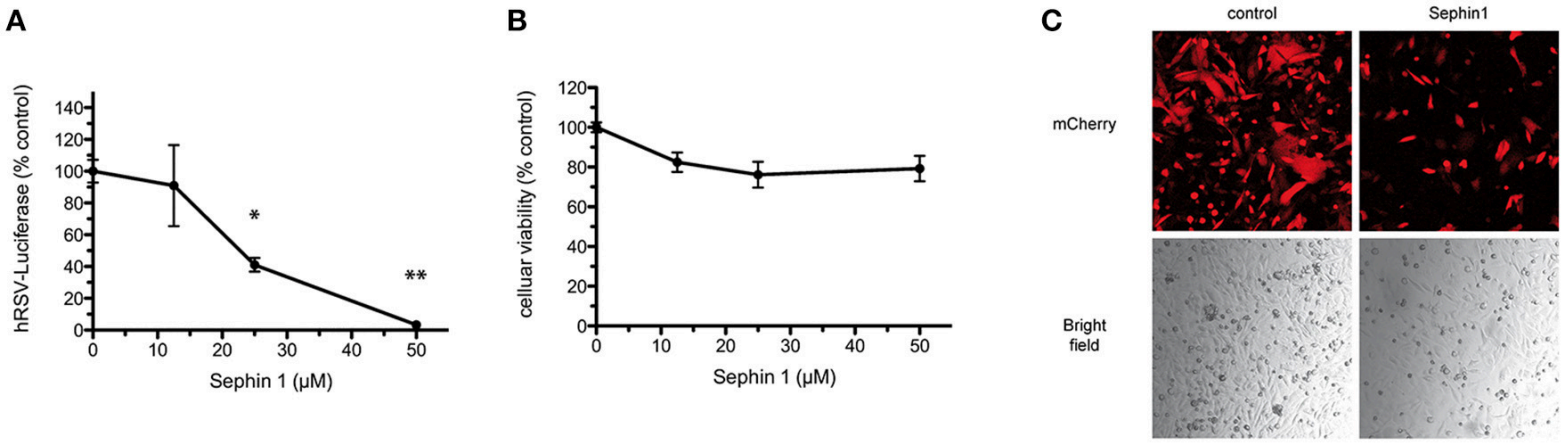
Evaluation of the antiviral properties of Sephin1 against hRSV. **(A)** HEp-2 cells were infected with a recombinant strain of hRSV expressing luciferase and incubated with increasing doses of Sephin1 or DMSO alone. After 24 h, luciferase expression was determined. **(B)** Viability of HEp-2 cells incubated with increasing doses of Sephin1 or DMSO alone was determined after 24 h of incubation using the cellular viability assay Vita-Blue. **(C)** HEp-2 cells were infected with a recombinant strain of hRSV expressing mCherry and incubated with increasing doses of Sephin1 or DMSO alone. After 24 h, cells were fixed and imaged using a confocal microscope. Data represent mean ± SEM from representative experiments, repeated at least three times. ^*^p < 0.05, ^**^p < 0.01 by one-way analysis of variance, followed by Bonferroni comparison test comparing the Sephin1-treated group at the indicated concentration to the vehicle-treated control group.

Human respiratory syncytial virus (hRSV) is a negative strand RNA virus belonging to the *Pneumoviridae* family. hRSV is a common cause of acute lower respiratory disease in infants and young children. Current antiviral therapies against hRSV are limited to an expensive humanized monoclonal antibody used as a prophylactic treatment and to ribavirin, which has limited efficacy and relatively high toxicity (42). There is therefore a need to develop new antiviral therapies. In order to test if Sephin1 was able to inhibit hRSV replication, we infected HEp-2 cells with a genetically engineered hRSV expressing the Firefly luciferase, used to quantify virus replication (24). Following virus adsorption for 1 h, cells were treated with increasing doses of Sephin1. Measurement of luciferase activity 24 h post-infection revealed a dose dependent inhibition of hRSV replication by Sephin1 (Figure 2A). A 30-fold inhibition of replication was observed when Sephin1 was used at 50 μM, which is the highest dose used in the experiments. Cellular viability was measured with a fluorescent dehydrogenase enzymes substrate, which reveals cellular metabolic activity. Cellular viability did not decrease significantly following Sephin1 treatment for 24 h and remained above 80% in HEp-2 cells treated with 50 μM Sephin1 (Figure 2B). Fluorescence and bright-field microscopic analysis of HEp-2 cells infected with a genetically engineered hRSV expressing the fluorescent protein Cherry (24) confirmed that treatment with 50 μM Sephin1 for 24 h led to a significant reduction of virus replication and was not associated with significant changes in cellular morphology or density (Figure 2C).

**Figure 3:**
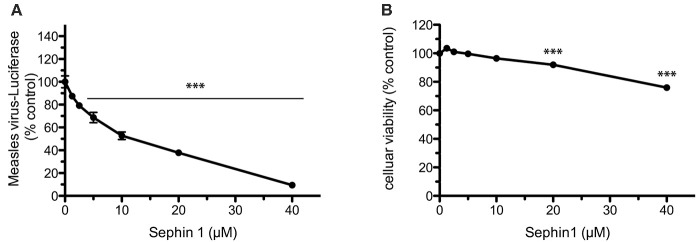
Evaluation of the antiviral properties of Sephin1 against measles virus. **(A)** HEK293T cells were infected with a recombinant strain of measles virus expressing luciferase and incubated with increasing doses of Sephin1 or DMSO alone. After 24 h, luciferase expression was determined. Data represent mean values from a representative experiment, repeated at least three times. **(B)** Viability of HEK293T cells incubated with increasing doses of Sephin1 or DMSO alone was determined after 24 h incubation using the cellular viability assay Vita-Blue. Data represent mean ± SEM from representative experiments, repeated at least three times. ^***^p < 0.001 by one-way analysis of variance, followed by Bonferroni comparison test comparing the Sephin1-treated groups at the indicated concentrations to the vehicle-treated control group.

We further documented the antiviral spectrum of Sephin1 by testing its antiviral potential against measles virus. Measles virus is a negative strand RNA virus belonging to the *Paramyxoviridae* family currently causing large outbreaks due to suboptimal vaccination coverage in many countries (43). To evaluate the antiviral properties of Sephin1 against measles virus, we infected human HEK293T cells with a genetically engineered measles virus expressing the Firefly luciferase (28). Following virus adsorption for 1 h, cells were treated with increasing doses of Sephin1. Measurement of luciferase activity 24 h post-infection revealed a dose dependent inhibition of measles virus replication in HEK293T cells by Sephin1 (Figure 3A). A 10-fold inhibition of replication was observed when Sephin1 was used at 40 μM, which is the highest dose used in these experiments. Cellular viability remained above 75% in HEK293T cells treated for 24 h with 40 μM Sephin1 (Figure 3B).

**Figure 4:**
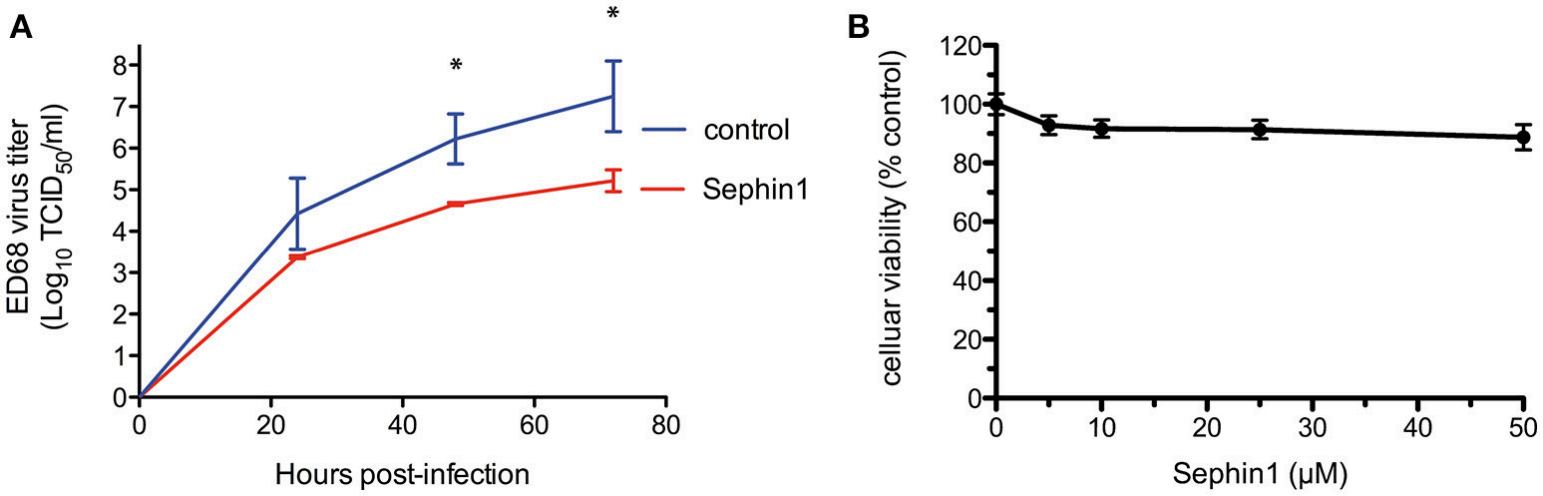
Evaluation of the antiviral properties of Sephin1 against enterovirus ED68. **(A)** RD cells were infected with enterovirus ED68 and incubated at 33°C in the presence of 50 μM Sephin1 or DMSO alone (control). Viral titers were determined from supernatants harvested at the indicated times post-infection. **(B)** Viability of RD cells incubated with increasing doses of Sephin1 or DMSO alone was determined after 24 h of incubation using the cellular viability assay Vita-Blue. Data represent mean ± SEM from representative experiments, repeated at least three times. ^*^p < 0.05 by one-way analysis of variance, followed by Bonferroni comparison test comparing the Sephin1-treated groups at the indicated concentrations to the vehicle-treated control group.

We next tested the antiviral properties of Sephin1 against enterovirus D68. Enterovirus D68 is a positive strand RNA virus belonging to the *Picornaviridae* family and causing upper respiratory tract infections in children (44). To mimic physiological temperatures found in the human upper tract, we infected human RD cells grown at 33°C with enterovirus D68 (26). Cells were infected at a low MOI to allow multiple cycles of infection. Following virus adsorption for 1 h, cells were treated with 50 μM Sephin1 and supernatants collected at the indicated time post-infection to quantify viral load by standard tissue culture infectious dose 50 (TCID_50_) method. Treatment with 50 μM Sephin1 caused a more than 10-fold reduction of enterovirus ED68 titers (Figure 4A). Inhibition of enterovirus ED68 was readily detected at 24 h post-infection and persisted throughout the experiment up to 72 h post-infection, even though Sephin1 was added via a single treatment in the culture medium at 1 h post-infection. RD cells viability did not decrease significantly following 50 μM Sephin1 treatment for 24 h and remained above 90% (Figure 4B), consistent with cellular viability results observed in Sephin1-treated HEp-2 cells and HEK293T cells.

**Figure 5:**
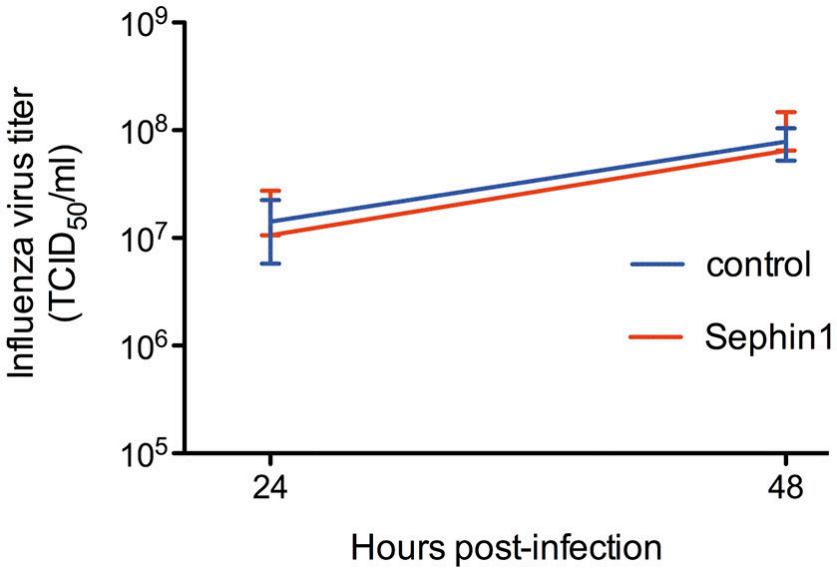
Evaluation of the antiviral properties of Sephin1 against influenza virus. A549 cells were infected with influenza A/Puerto Rico/8/1934 (H1N1) strain and incubated in the presence of 50 μM Sephin1 or DMSO alone (control). Viral titers were determined from supernatants harvested at the indicated times post-infection. Data represent mean ± SEM from a representative experiment, repeated at least three times.

We further tested the antiviral potential of Sephin1 against influenza A virus. We infected human A549 cells with the influenza A/Puerto Rico/8/1934 (H1N1) strain at a low multiplicity of infection. Following 1 h adsorption, A549 cells were treated with 50 μM Sephin1 or control cells treated with vehicle only. Viral titers in the supernatants were determined by standard plaque assay. We did not observe any inhibitory effect of Sephin1 on influenza virus replication (Figure 5). Similar results were obtained when experiments were performed on the canine MDCK cell line or when experiments were performed with the avian influenza A/turkey/Italy/977/1999(H7N1) virus strain (data not shown).

**Figure 6:**
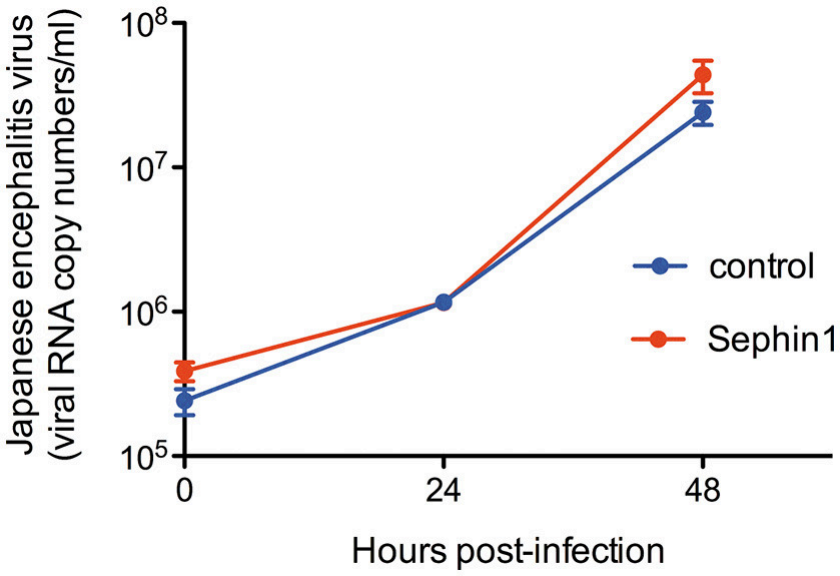
Evaluation of the antiviral properties of Sephin1 against Japanese encephalitis virus. HEK293T cells were infected with Japanese encephalitis virus and incubated in the presence of 50 μM Sephin1 or DMSO alone (control). Viral titers were determined by quantitative RT-PCR from supernatants harvested at the indicated times post-infection. Data represent mean ± SEM from a representative experiment, repeated at least three times.

To assess the antiviral potential of Sephin1 against a virus belonging to the *Flaviviridae* family, we infected HEK293T cells with Japanese encephalitis virus. Sephin 1 had no effect on the replication of Japanese encephalitis virus, as determined by quantifying viral genomes in the supernatants by quantitative RT-PCR (Figure 6). We thus identified viruses that are not inhibited by Sephin1, demonstrating that although Sephin1 has a broad antiviral spectrum, it is not active against all viruses.

### Evaluation of the Antiviral Properties of Sephin1 Against DNA Viruses

**Figure 7:**
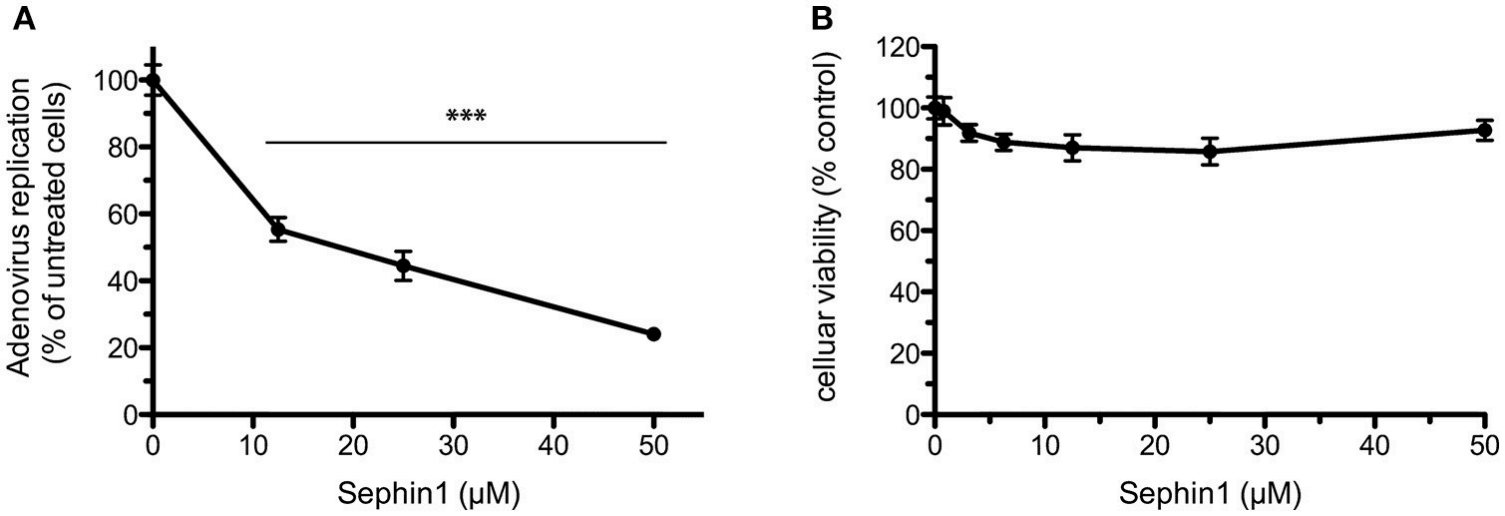
Evaluation of the antiviral properties of Sephin1 against human Adenovirus. **(A)** HEK293 cells were infected with a recombinant strain of hAdV expressing ANCHOR3 and incubated with increasing doses of Sephin1 or DMSO alone. After 24 h, virus replication was determined by automated counting of GFP foci. **(B)** Viability of HEK293 cells incubated with increasing doses of Sephin1 or DMSO alone was determined after 24 h incubation using the cellular viability assay Vita-Blue. Data represent mean ± SEM from representative experiments, repeated at least three times. ^***^p < 0.001 by one-way analysis of variance, followed by Bonferroni comparison test comparing the Sephin1-treated groups at the indicated concentrations to the vehicle-treated control group.

In order to test if Sephin1 could inhibit phylogenetically distant viruses, we analyzed its antiviral potential against human Adenovirus (hAdV), a DNA virus belonging to the *Adenoviridae* family causing respiratory tract infections in humans. A genetically modified hAdV expressing the bacterial partitioning system-based AnchOR3 was used to infect HEK293 cells. The AnchOR3 system allows for the real-time detection of viral DNA replication in living cells through the detection of GFP foci and is therefore used to monitor DNA virus replication in real-time by fluorescent microscopy (27). Cells were treated with increasing doses of Sephin1 or vehicle only and infected immediately with AnchOR3 hAdV. Measurement of GFP fluorescent foci by automated microscopy 24 h post-infection revealed a dose dependent inhibition of hAdV replication by Sephin1 (Figure 7A). A four-fold inhibition of replication was observed when Sephin1 was used at 50 μM, which is the highest dose used in the experiments. Cellular viability did not decrease significantly following Sephin1 treatment for 24 h and remained above 80% in HEK293 cells treated with 50 μM Sephin1 (Figure 7B).

**Figure 8:**
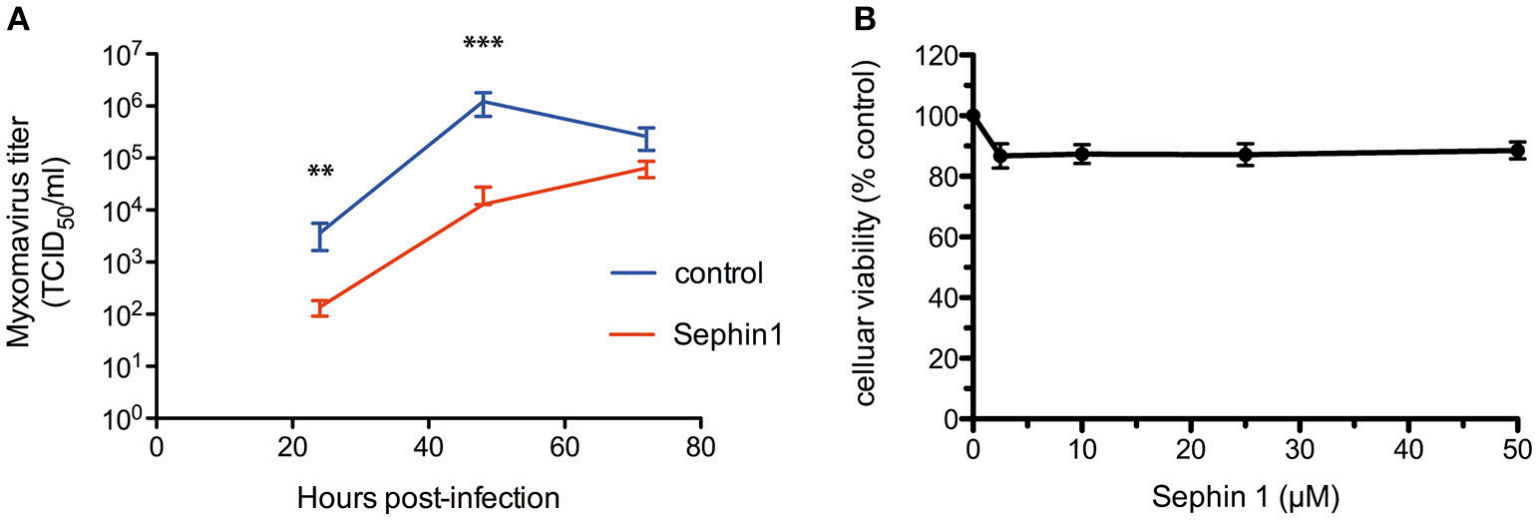
Evaluation of the antiviral properties of Sephin1 against myxoma virus. **(A)** RK13 cells were infected with myxoma virus and incubated in the presence of 50 μM Sephin1 or DMSO alone (control). Viral titers were determined from crude lysates harvested at the indicated times post-infection. **(B)** Viability of RK13 cells incubated with increasing doses of Sephin1 or DMSO alone was determined after 24 h incubation using the cellular viability assay Vita-Blue. Data represent mean ± SEM from representative experiments, repeated at least three times. ^**^p < 0.001, ^***^p < 0.001 by one-way analysis of variance, followed by Bonferroni comparison test comparing the Sephin1-treated groups at the indicated concentrations to the vehicle-treated control group.

We next analyzed the antiviral potential of Sephin1 against myxoma virus, a DNA virus of the *Poxviridae* family, which contains pathogens of major importance in human and veterinary medicine. Myxoma virus is responsible for Myxomatosis in European rabbits, a disease of medical importance in veterinary medicine, worsened due to the emergence of strains causing respiratory diseases in rabbits (39). Following myxoma virus adsorption for 1 h, rabbit RK13 cells were treated with 50 μM Sephin1. Cells and supernatants were harvested at 24, 72, and 120 h post-infection and subjected to three freeze-thaw cycles to detect free viral particles, as well as cell-associated virus particles (30). Virus titration by standard plaque-assay revealed that Sephin1 significantly inhibited myxoma virus replication (Figure 8A). Cellular viability did not decrease significantly following Sephin1 treatment of RK13 cells for 24 h (Figure 8B).

**Figure 9:**
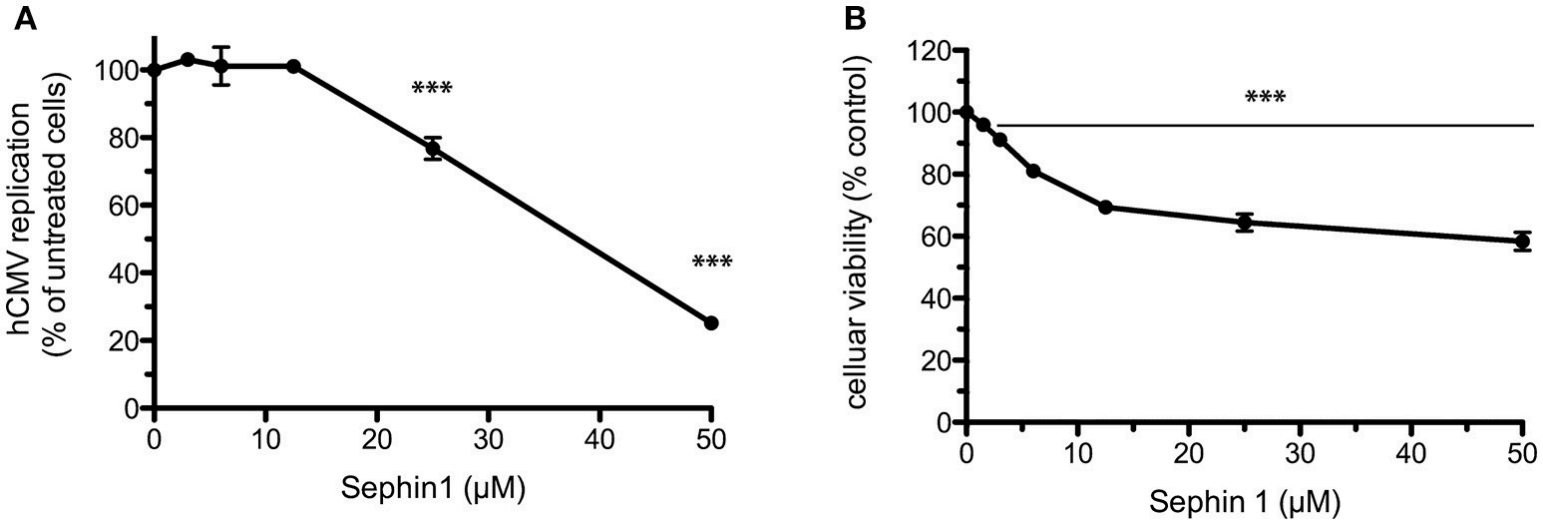
Evaluation of the antiviral properties of Sephin1 against human cytomegalovirus. **(A)** ARPE-19 cells were infected with a recombinant strain of hCMV expressing ANCHOR3 and incubated with increasing doses of Sephin1 or DMSO alone. After 72 h, virus replication was determined by automated counting of GFP foci. **(B)** Viability of ARPE-19 cells incubated with increasing doses of Sephin1 or DMSO alone was determined after 72 h incubation using the cellular viability assay Vita-Blue. Data represent mean ± SEM from representative experiments, repeated at least three times. ^***^p < 0.001 by one-way analysis of variance, followed by Bonferroni comparison test comparing the Sephin1-treated groups at the indicated concentrations to the vehicle-treated control group.

Finally, we evaluated the antiviral of Sephin1 against human cytomegalovirus (hCMV), a DNA virus belonging to the *Herpesviridae* family. hCMV is widespread in the human population and causes severe diseases following congenital infection. A genetically modified hCMV expressing the AnchOR3 system to detect viral DNA replication by the accumulation of GFP foci was used to infect the human retinal pigment cell line ARPE-19 (31). Sephin1 had a dose-dependent inhibitory effect on hCMV replication in the human ARPE-19 cell line, reaching a five-fold inhibition at 50 μM (Figure 9A). At this dose Sephin1 caused a 40% reduction in cellular viability, indicating that Sephin1 is associated with moderate toxicity at 50 μM in the ARPE-19 cell line (Figure 9B). Altogether these results provide evidence that Sephin1 has antiviral activity against respiratory viruses belonging to phylogenetically distant families.

### Evaluation of the Contribution of eIF2α Phosphorylation to the Antiviral Effects of Sephin1

**Figure 10:**
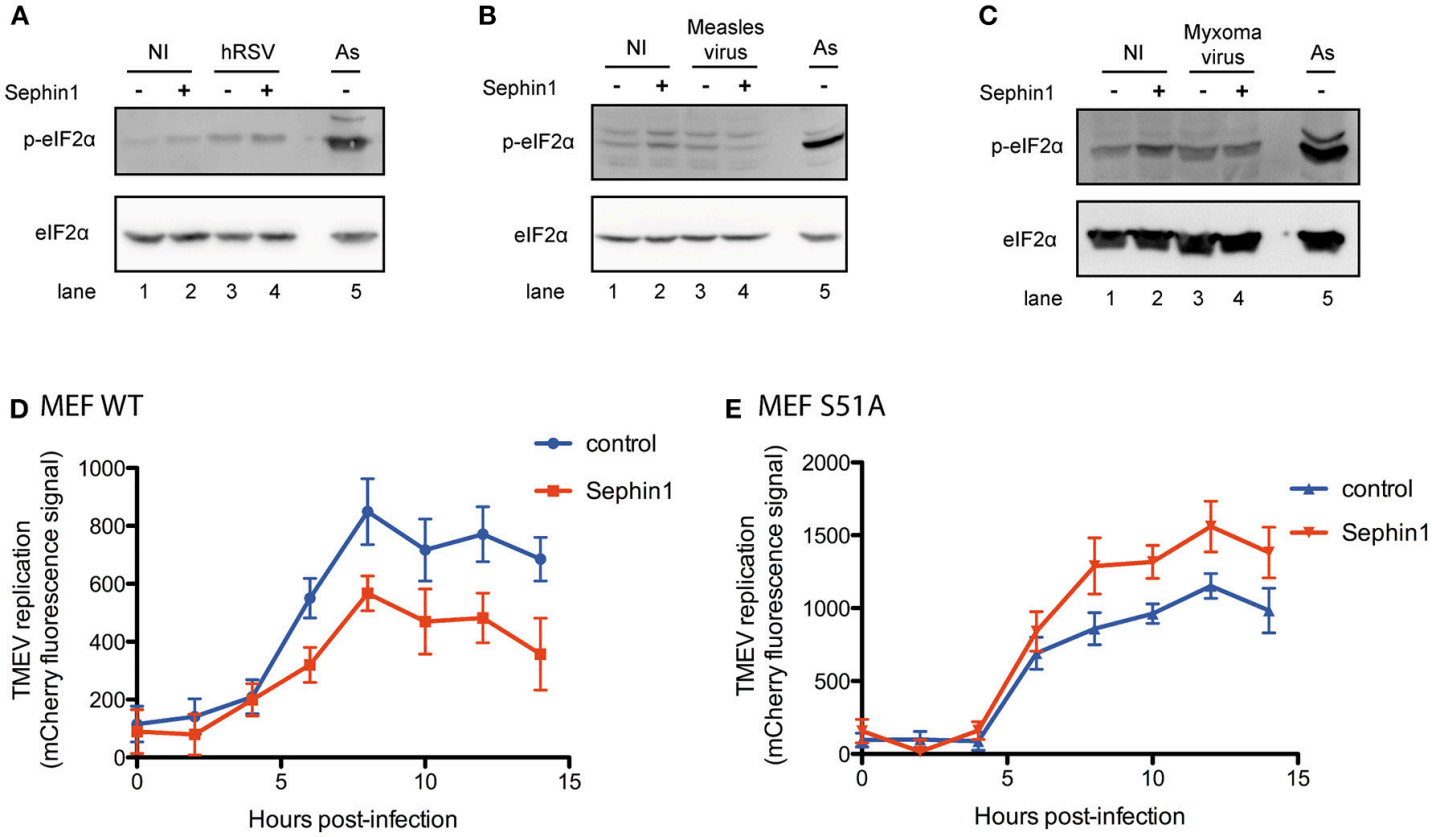
Evaluation of the contribution of eIF2α phosphorylation to the antiviral effects of Sephin1. Cells were either left non-infected (NI) or infected with the indicated viruses, in the presence or absence of 50 μM Sephin1. Sodium arsenite (As), a potent inducer of eIF2α phosphorylation, was used as a positive control for detection of eIF2α phosphorylation by western-blot. **(A)** Analysis of eIF2α phosphorylation in HEp-2 cells infected with hRSV. **(B)** Analysis of eIF2α phosphorylation in HEK293T cells infected with measles virus. **(C)** Analysis of eIF2α phosphorylation in RK13 cells infected with myxoma virus. **(D)** Consequences of Sephin1 treatment on TMEV replication in WT mouse embryonic fibroblasts (MEF WT). **(E)** Consequences of Sephin1 treatment on TMEV replication in mouse embryonic fibroblasts expressing a non-phosphorylable (S51A) allele of eIF2α (MEF S51A). TMEV replication was determined by measuring mCherry fluorescence. Data represent mean ± SEM from representative experiments, repeated at least three times.

To test if the antiviral effect of Sephin1 correlated with increased eIF2α phosphorylation, we performed western-blot analyses of virus-infected cells. eIF2α phosphorylation was increased in cells infected with hRSV (Figure 10A, compare lanes 1 and 3). However, treatment with 50 μM Sephin1 did not increase eIF2α phosphorylation in hRSV-infected cells (Figure 10A, compare lanes 3 and 4). eIF2α phosphorylation was not increased in cells infected with measles virus (Figure 10B, compare lanes 1 and 3) or in cells infected with myxoma virus (Figure 10C, compare lanes 1 and 3), even when cells were treated with 50 μM Sephin1 (Figures 10B,C, compare lanes 3 and 4). Thus, the antiviral activity of Sephin1 does not correlate with increased eIF2α phosphorylation, raising the possibility that some antiviral effects of Sephin1 could be independent of eIF2α phosphorylation.

To test if Sephin1 could inhibit virus replication independently of eIF2α phosphorylation, we compared wild-type mouse embryonic fibroblasts (MEF WT) and mouse embryonic in which the endogenous eIF2α gene has been genetically replaced by a nonphosphorylable (S51A) allele (MEF S51A) (22). These cells were infected with Theiler's murine encephalomyelitis virus (TMEV), a positive strand RNA virus belonging to the *Picornaviridae* family. We used a genetically modified virus, which has a deleted L protein, rendering the virus highly susceptible to the antiviral effects of PKR and expressing the fluorescent protein Cherry, used as a reporter to quantify virus replication (36). Following virus adsorption for 1 h, cells were either treated with vehicle only or treated with 50 μM Sephin1. Virus replication was evaluated by measuring Cherry fluorescence using a fluorescent microplate reader. Sephin1 reduced Cherry fluorescence in MEF WT cells, indicating that it inhibited TMEV-Cherry replication in MEF WT cells (Figure 10D). Surprisingly, Cherry fluorescence was higher in Sephin1-treated MEF S51A cells compared to non-treated S51A cells, indicating that Sephin1 treatment increased TMEV replication in cells expressing nonphosphorylable eIF2α (Figure 10E). It is currently unclear how Sephin1 could increase TMEV replication in cells expressing nonphosphorylable eIF2α. This result nevertheless demonstrates that eIF2α phosphorylation is required for the antiviral effects of Sephin1 against TMEV.

### Sephin1 Is Showing Some Antiviral Effect *in vivo*, Which Is However Limited by Toxic Side Effects

**Figure 11:**
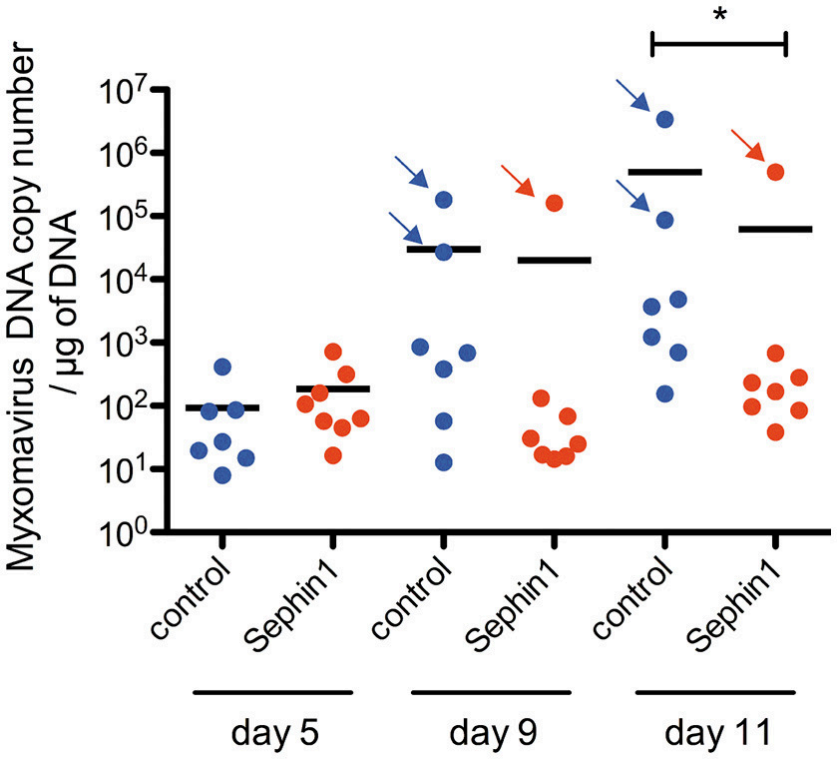
Evaluation of the antiviral potential of Sephin1 against myxoma virus *in vivo*. Rabbits were inoculated with 50 plaque-forming units of myxoma virus. Eight rabbits were treated with Sephin1 by oral gavage once daily at a dose of 5 mg/kg. Seven rabbits were administered vehicle only. Myxoma virus DNA was detected by q-PCR from conjonctival swabs performed at the indicated days post-infection. Rabbits exhibiting clinical signs are indicated with an arrow. ^*^p < 0.05 by the two-tailed Mann–Whitney test.

In order to evaluate if Sephin1 could exert antiviral activity *in vivo*, we evaluated its therapeutic potential in European rabbits infected with myxoma virus. European rabbits are the natural hosts of myxoma virus. Rabbits were inoculated with 50 plaque-forming units of myxoma virus strain LH 3082 by intradermal inoculation in the right ear lobe. Sephin1 was administered by oral gavage once daily at a dose of 5 mg/kg. Treatment with Sephin1 began straight after virus inoculation. Control rabbits were treated similarly with vehicle. Except for two rabbits in the control group and one rabbit in the Sephin1-treated group, no other infected rabbits developed clinical signs of myxomatosis over the period of observation. Sephin1 appeared to be well-tolerated at a daily dose of 5mg/kg, as Sephin1-treated rabbits were clinically indistinguishable from the control rabbits. Conjonctival swabs were performed on day 0, 5, 9, and 11 to monitor for virus replication by q-PCR (Figure 11). Levels of viral DNA increased in non-treated control animals over the observation period, indicating efficient virus replication in the rabbits. The levels of virus replication were much higher for the three rabbits showing clinical signs, indicated by arrows in Figure 11. We observed a significant reduction in virus replication at day 11 post-infection in the Sephin1-treated group compared to the control group, demonstrating that Sephin1 can exert an antiviral activity *in vivo*. However, the antiviral activity of Sephin1 given orally at 5 mg/kg daily was modest. We therefore repeated the experiment in order to administer Sephin1 at a higher dosage. In this second *in vivo* experiment, rabbits were infected as previously. Sephin1 was administered by oral gavage once daily at a dose of 100 mg/kg beginning straight after inoculation. When used at 100 mg/kg, acute toxicity was observed as soon as 2 days post-infection in the Sephin1 treated rabbits, which developed anorexia and presented ruffled fur. For ethical reasons, in compliance with the guidelines from the animal care and use committee, we euthanized the animals and terminated the experiment. Altogether these results suggest that although Sephin1 has some antiviral activity *in vivo* at 5 mg/kg, increasing the dosage to reach higher concentrations and possibly better antiviral activity is currently not possible due to the existence of major side effects.

## Discussion

Negative strand RNA viruses, positive strand RNA viruses, as well as DNA viruses were inhibited by Sephin1 treatment in cell culture. Our results thus provide evidence that Sephin1 treatment has antiviral properties against a broad range of viruses belonging to phylogenetically distant viral families. A four to 100-fold inhibition of viral replication was obtained when Sephin1 was used at 50 μM. At this dose, cellular viability remained above 75% in all cell lines tested, with the exception of ARPE-19 cells, which had a 40% decrease in viability. This result demonstrates that, although the molecule had to be used at a high dose to reach a significant antiviral effect, inhibition of virus replication could not be attributable to alterations in cellular viability.

Secondary structures found in the RNA of some positive strand RNA viruses, such as viruses belonging to the *Togaviridae* family, *Reoviridae* family and hepatitis C virus, allow translation of these RNAs to proceed normally, or in some cases better, in the presence of high levels of eIF2α phosphorylation (12). It is therefore expected that Sephin1 would have no antiviral effect against these viruses. In the case of influenza virus, the lack of antiviral activity of Sephin1 might be attributable to the tight inhibition of PKR activation by the viral protein NS1 (45). This inhibition is mediated by the binding of NS1 to PKR (46). Influenza A viruses with mutant NS1 proteins unable to bind to PKR are highly attenuated in wild-type mice, but replicate to high levels in PKR deficient mice (46). By contrast, wild-type influenza viruses replicate to similar levels in wild-type mice and in PKR deficient mice (46, 47). These observations suggest that PKR is an important antiviral pathway against influenza viruses, which is very efficiently counteracted by wild-type NS1 protein. The lack of activity of Sephin1 observed in cell culture may be due to the absence of PKR activation and eIF2α phosphorylation in influenza virus infected cells, as previously described (48). Similarly, the lack of antiviral activity of Sephin1 against Japanese encephalitis virus could be due to the tight inhibition of PKR activation by the viral protein NS2A (49). In the absence of eIF2α phosphorylation and consequent expression of GADD34, it is anticipated that Sephin1 would have no effect.

GADD34 has been shown to stimulate type I interferon production in response to the synthetic viral RNA analog poly(I:C) and in response to infection with Chikungunya virus, a member of the *Togaviridae* family (50). Activation of PKR by poly(I:C) and in response to Chikungunya virus infection leads to eIF2α phosphorylation, which inhibits initiation of protein translation, including translation of type I interferons. GADD34 expression and subsequent dephosphorylation of eIF2α resume initiation of protein translation and consequently allow translation of type I interferons (50). Inhibition of GADD34 is therefore a double-edged sword. On one hand, increased eIF2α phosphorylation caused by inhibition of GADD34 can potentiate the antiviral effects by causing a tighter inhibition of viral protein translation. On the other hand, a prolonged increase of eIF2α phosphorylation caused by inhibition of GADD34 can inhibit the translation of host proteins involved in antiviral defense, such as type I interferons and antiviral effector proteins. The potential beneficial effects of GADD34 inhibition are difficult to predict. For viruses, such as members of the *Togaviridae*, which are able to translate their proteins in the presence of high levels of eIF2α phosphorylation, inhibition of GADD34 will likely be detrimental to the host because of a reduction in the translation of host proteins involved in antiviral defense. For viruses, which are unable to translate their proteins in the presence of high levels of eIF2α phosphorylation, the consequences of GADD34 inhibition on viral replication are to our knowledge not predictable, and therefore most likely need to be experimentally tested.

Inhibition of myxoma virus and measles virus by Sephin1 was not associated with increased levels of eIF2α phosphorylation. We thus did not observe a strict correlation between the antiviral effects of Sephin1 and eIF2α phosphorylation. One tentative explanation is that viral inhibition leads to a reduction in the levels of viral PKR activators in Sephin1-treated cells compared to infected non-treated cells. However, these observations also raise the possibility that Sephin1 does not act by targeting GADD34-PP1 mediated dephosphorylation of eIF2α. Indeed, contradicting the initial description (18) and follow-up work (21), Sephin1 and its derivative guanabenz were recently shown to lack any effect on GADD34-PP1 mediated dephosphorylation of eIF2α (19, 20, 51). We cannot rule out that Sephin1 mediates its effects independently of GADD34. However, we observed increased phosphorylation of eIF2α in cells stimulated with the PERK activator tunicamycin and in cells stimulated with the PKR activator poly(I:C). Moreover, the lack of antiviral activity of Sephin1 against Theiler's murine encephalomyelitis virus (TMEV) in MEF cells expressing a nonphosphorylable (S51A) allele demonstrates that eIF2α phosphorylation is required for the antiviral effects of Sephin1 against TMEV. Whether these effects are due to a specific inhibition of GADD34 by Sephin1 remains to be investigated.

We observed a modest antiviral effect of Sephin1 administered by oral gavage once daily at a dose of 5 mg/kg against myxoma virus in rabbits. At 5 mg/kg, no toxic side effects were detected by clinical examination of the rabbits. However, when we administered Sephin1 by oral gavage once daily at a dose of 100 mg/kg, major clinical signs were detected, indicating that at this dosage Sephin1 caused acute toxicity. GADD34 knock-out mice were viable and did not show any clinical signs under normal breeding conditions (52). This finding suggests that the toxic side effects of Sephin1 observed in rabbits are unlikely due to inhibition of GADD34, but rather point to Sephin1-induced alterations in physiology that are independent of GADD34. Ongoing studies to identify the causes of toxicity could provide information for the development of new treatment regimens, including new formulations and modes of administration. In addition, Sephin1 can be the scaffold of structure-activity relationship studies to identify new variants with increased efficiency or decreased *in vivo* toxicity, and thus exhibiting an improved selectivity index to consider these new variants as promising therapeutic antiviral candidates. In this chemical series, this is already well exemplified by the development of Sephin1 itself, which is derived from guanabenz to eliminate some unwanted binding to the α2- adrenergic receptor (18).

The prominent role of the PKR eIF2α pathway in antiviral defense is well-established. Direct stimulators of PKR will stimulate eIF2α phosphorylation in all cells exposed to the drug, therefore likely leading to unwanted side effects in non-infected cells. By contrast, GADD34 expression is stress-inducible and drugs targeting GADD34 should therefore be active only in cells that have increased levels of eIF2α phosphorylation, including virus-infected cells, thus increasing selectivity. GADD34 inhibitors would most likely be most effective in complement with other molecules, such as drugs targeting viral PKR antagonists or drugs thought to affect viral protein folding, such as nitazoxanide (53) or iminosugars (54), which could potentiate PERK-mediated eIF2α phosphorylation in infected cells.

## Author Contributions

MF-B, GD, PB, SK, CQ-F, CB, SL, SB, P-OV, FG, and RV performed the experiments and analyzed the data. CB, SL, M-AR-W, J-FE, FT, BL, and FG provided reagents and analyzed the data. RV drafted the manuscript. All the authors contributed to the critical review and revision of the manuscript.

### Conflict of Interest Statement

The authors declare that the research was conducted in the absence of any commercial or financial relationships that could be construed as a potential conflict of interest.
