# Supplementary figures and images for: Evaluation of the Antiviral Activity of Sephin1 Treatment and Its Consequences on eIF2α Phosphorylation in Response to Viral Infections
Source: Front Immunol. 2019 Feb 12;10:134. doi: 10.3389/fimmu.2019.00134 (PMC6379315; doi:10.3389/fimmu.2019.00134)

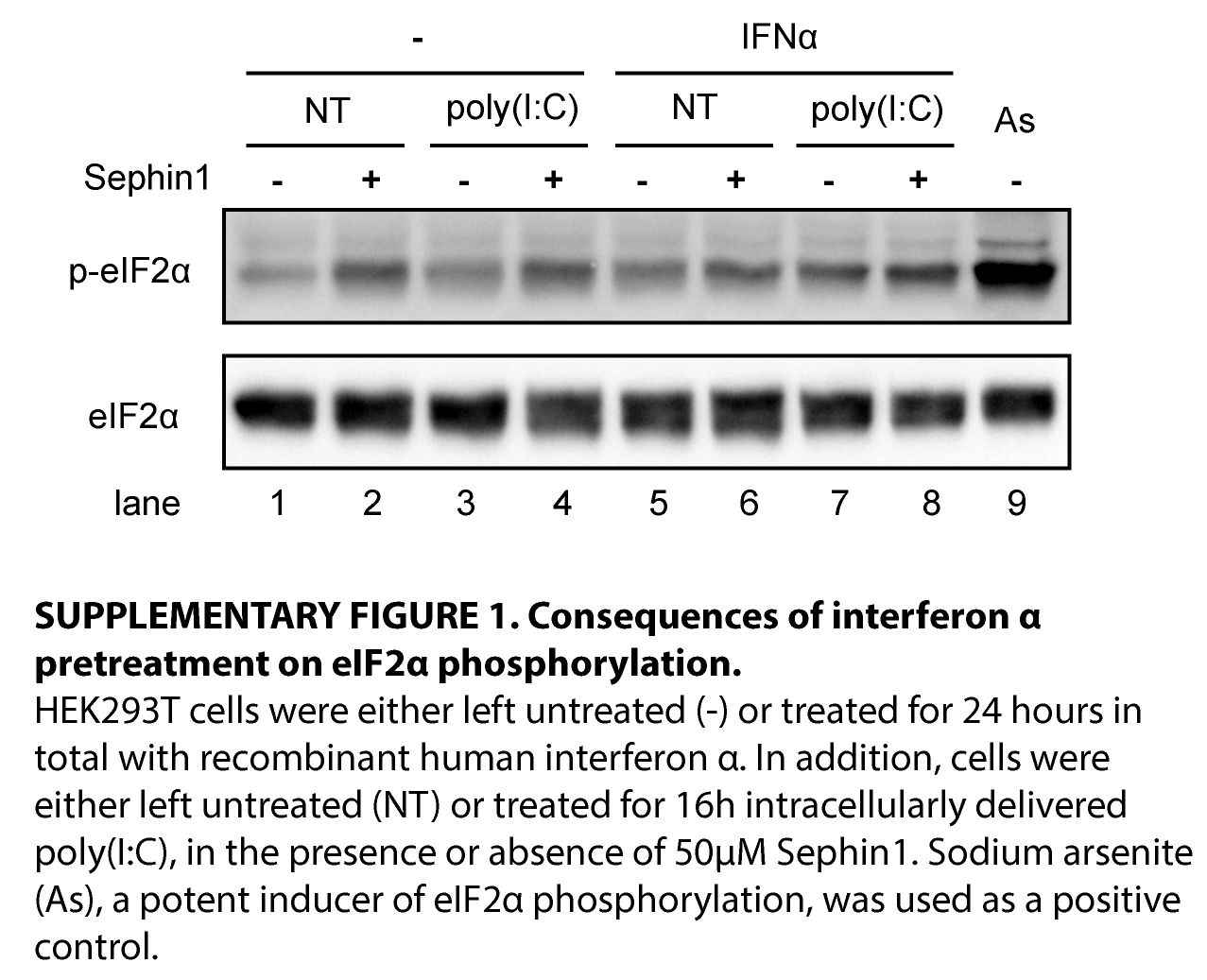

Supplement: Supplementary file 1 [file Image_1.TIF]
